# Extraosseous Ewing sarcoma of the distal pancreas: diagnostic pitfalls and the critical role of molecular testing

**DOI:** 10.1093/jscr/rjag295

**Published:** 2026-04-23

**Authors:** Rakesh Kumar Singh, Tushar Saini, Sanjay Kumar, Kumar Kaushik, Vinaysheel Priyadarshi

**Affiliations:** Department of Surgical Gastroenterology, Indira Gandhi Institute of Medical Sciences, Bailey Road, Raja Bazar, Patna District, Patna 800014, Bihar, India; Department of General Surgery (GI Surgery Unit), Rohilkhand Medical College and Hospital, Pilibhit Bypass Road (Pawan Vihar / Mahanagar area), Bareilly District, Bareilly 243006, Uttar Pradesh, India; Department of Surgical Gastroenterology, Indira Gandhi Institute of Medical Sciences, Bailey Road, Raja Bazar, Patna District, Patna 800014, Bihar, India; Department of General Surgery, Rohilkhand Medical College and Hospital, Pilibhit Bypass Road (Pawan Vihar / Mahanagar area), Bareilly District, Bareilly 243006, Uttar Pradesh, India; Department of General Surgery, Rohilkhand Medical College and Hospital, Pilibhit Bypass Road (Pawan Vihar / Mahanagar area), Bareilly District, Bareilly 243006, Uttar Pradesh, India

**Keywords:** pancreatic Ewing sarcoma, distal pancreatectomy, NKX2.2, ESFT, PNET

## Abstract

Primary pancreatic Ewing sarcoma (ES) is an exceedingly rare malignancy, with fewer than 100 confirmed cases reported worldwide. We report the case of a young adult woman presenting with a large solid-cystic pancreatic body-tail mass, initially diagnosed as solid pseudopapillary neoplasm and subsequently misinterpreted as metastatic endometrial stromal sarcoma following resection. Lack of adjuvant therapy and loss to follow-up preceded metastatic recurrence, whereupon targeted immunohistochemistry and molecular analysis established the definitive diagnosis of extraosseous ES with t(11;22)(q24;q12) translocation. Palliative chemotherapy yielded transient disease control before rapid progression and death. This case illustrates the diagnostic challenges posed by this rare entity due to overlapping clinicoradiologic and histologic features with more common pancreatic tumors, emphasizing the critical role of early inclusion of CD99, NKX2.2, and EWSR1 testing to facilitate appropriate multimodal management and improve outcomes.

## Introduction

Ewing sarcoma (ES) is an aggressive malignancy characterized by small, round, undifferentiated cells. It belongs to the Ewing sarcoma family of tumors (ESFT), encompassing osseous and extraosseous forms, and primarily affects children, adolescents, and young adults [1]. While the classic presentation involves bone, extraosseous ES can arise in soft tissues or visceral organs.

A hallmark of ESFT is the t(11;22)(q24;q12) translocation, resulting in the EWSR1-FLI1 fusion protein that promotes oncogenesis [2]. Diagnosis integrates histopathology (small round blue cells), immunohistochemistry (strong membranous CD99 and nuclear NKX2.2 positivity), and molecular confirmation of the characteristic translocation [3].

Primary pancreatic ES is exceedingly rare. Pancreatic malignancies are predominantly ductal adenocarcinomas in older adults, whereas primary pancreatic sarcomas constitute <0.1% of cases, with ES representing a minute subset [4, 5]. Systematic reviews through 2025 document only 51–89 confirmed primary pancreatic cases worldwide [4, 6]. This rarity frequently leads to initial misdiagnosis as more common entities, such as solid pseudopapillary neoplasm, neuroendocrine tumor, or pancreatitis-associated mass [7].

Accurate diagnosis is paramount, as ES management differs substantially from epithelial pancreatic cancers, requiring intensive multi-agent chemotherapy (typically vincristine, doxorubicin, and cyclophosphamide alternating with ifosfamide and etoposide), surgical resection when feasible, and radiotherapy as indicated [4]. Diagnostic delay or error adversely affects prognosis, particularly with metastatic progression. Herein, we report a case illustrating these diagnostic challenges in a young adult [8].

## Case report

A 28-year-old woman presented with upper abdominal pain, substantial weight loss (10 kg over 7 months), and a progressively enlarging abdominal mass noted over the preceding 2 months. Physical examination identified a large (~12 × 15 cm), hard, nodular, non-tender, fixed mass in the left upper quadrant.

**Figure 1:**
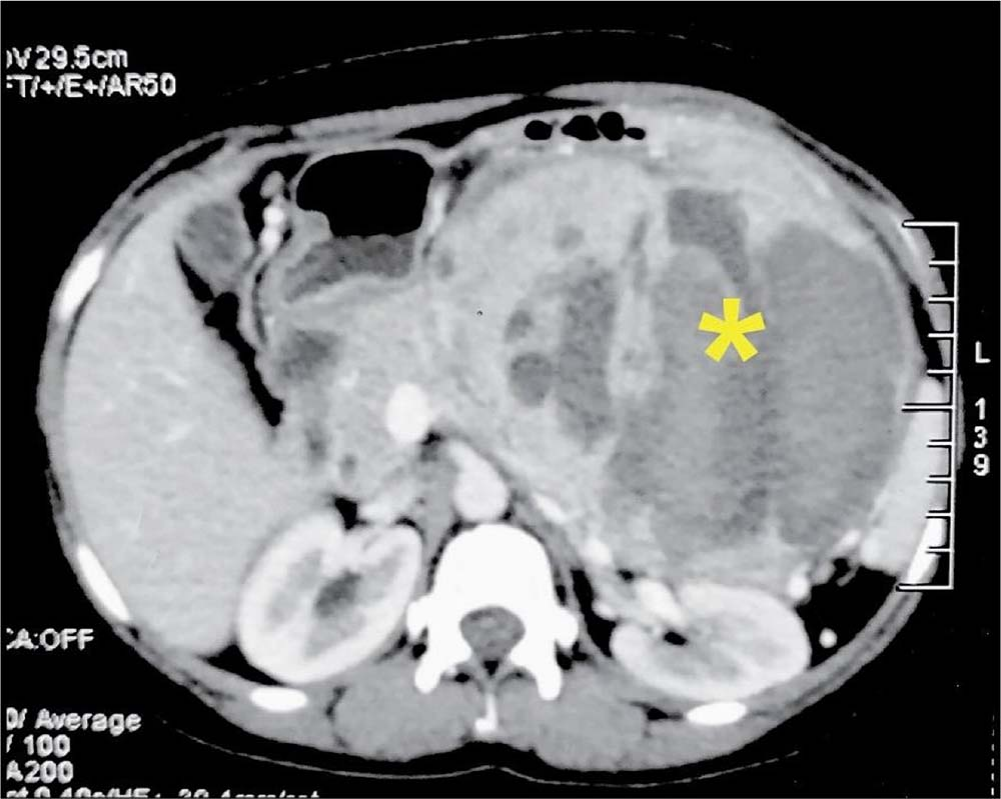
Preoperative CECT of the abdomen in the portal venous phase. The axial image demonstrates a large (14 × 11 × 19 cm) heterogeneously enhancing solid-cystic mass arising from the pancreatic body and tail. Key features include central necrosis (asterisk) and evidence of internal hemorrhage within the cystic components.

Contrast-enhanced computed tomography (CECT) of the abdomen revealed a 14 × 11 × 19 cm oval solid-cystic lesion originating from the pancreatic body and tail, with enhancing solid components and internal hemorrhage within cystic areas (Fig. 1). A provisional diagnosis of solid pseudopapillary neoplasm was favored, given its relative prevalence in young women.

**Figure 2:**
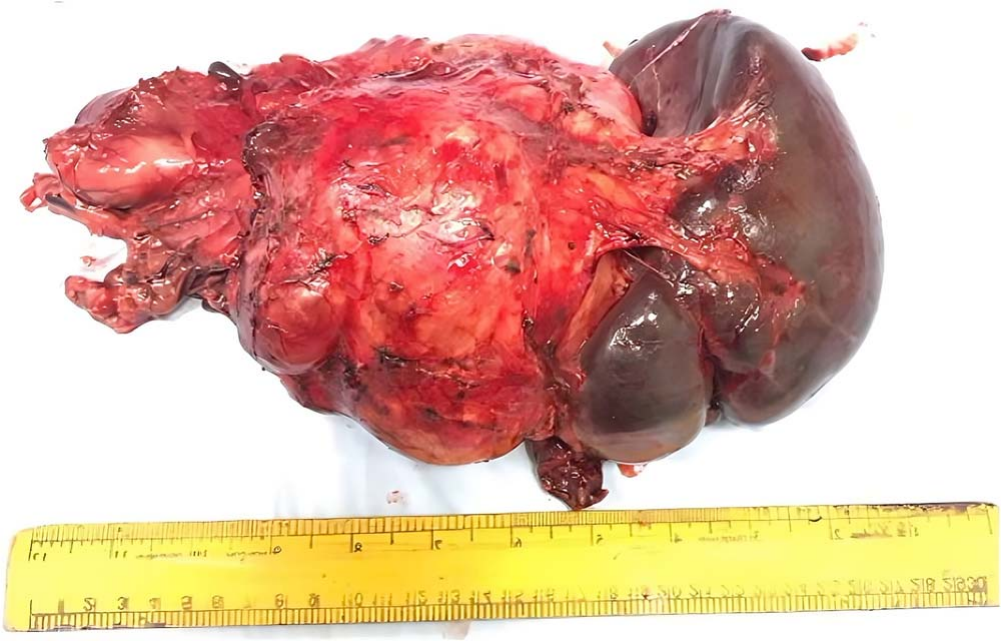
Gross photograph of the resected specimen. The image displays the distal pancreatectomy and splenectomy specimen, showing a large (15 × 12 cm), irregular mass arising from the pancreatic body and tail (left/center) en-bloc with the spleen (right). A ruler is provided at the bottom for scale reference.

Exploratory laparotomy disclosed a 15 × 12 cm mass arising from the pancreatic body and tail, with serosal infiltration of the posterior gastric wall and venous collaterals in the omentum and perisplenic region. Distal pancreatectomy with splenectomy (Fig. 2) achieved macroscopic complete resection, without evidence of lymphadenopathy or distant metastases.

**Figure 3:**
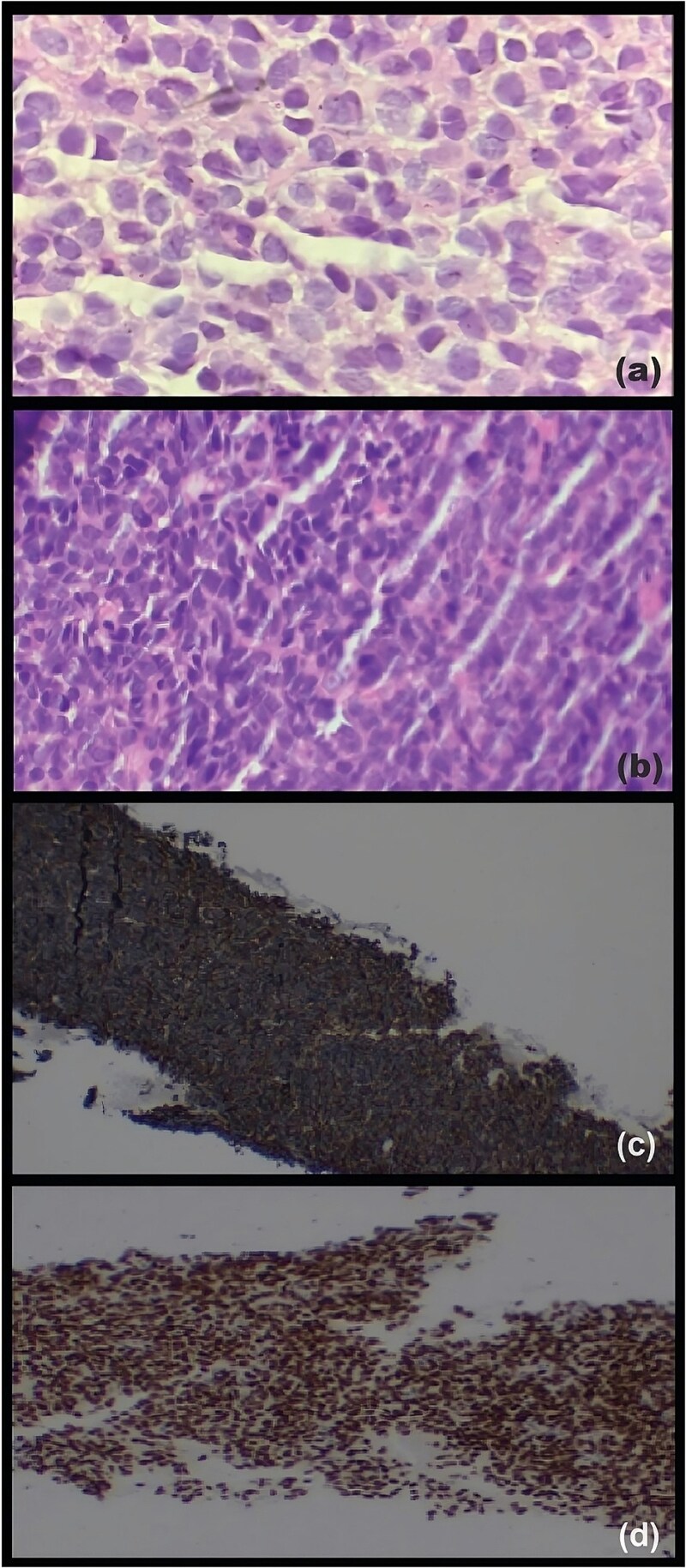
Histopathological and immunohistochemical findings. (a) Hematoxylin and eosin (H&E)-stained section of the primary pancreatic tumor showing diffuse sheets of small round cells with ovoid-to-folded nuclei, scant cytoplasm, indistinct nucleoli, and cystic spaces containing eosinophilic secretions (×200). (b) H&E-stained section of the ultrasound-guided liver metastasis biopsy revealing sheets of pleomorphic small round cells with coarse chromatin and a high nuclear-to-cytoplasmic ratio (×100). (c) Immunohistochemical staining of the metastatic liver lesion for CD99 demonstrating diffuse, strong membranous positivity (×20). (d) Immunohistochemical staining of the metastatic liver lesion for NKX2.2 demonstrating diffuse, strong nuclear positivity (×20).

Histopathological examination confirmed R0 resection with clear margins. Tumor was composed of diffuse sheets of small round cells with ovoid to folded nuclei, scant cytoplasm, indistinct nucleoli, and cystic spaces containing eosinophilic secretions (Fig. 3a). Immunohistochemistry showed positivity for pan-cytokeratin, S100, vimentin, CD10, cyclin D1, WT1, and SOX11, with negativity for synaptophysin, chromogranin, β-catenin, leukocyte common antigen, estrogen/progesterone receptors, CD117, DOG1, and BCL2. Based on morphology and immunoprofile, metastatic high-grade endometrial stromal sarcoma was provisionally diagnosed [9]. The patient was lost to follow-up without adjuvant therapy.

**Figure 4:**
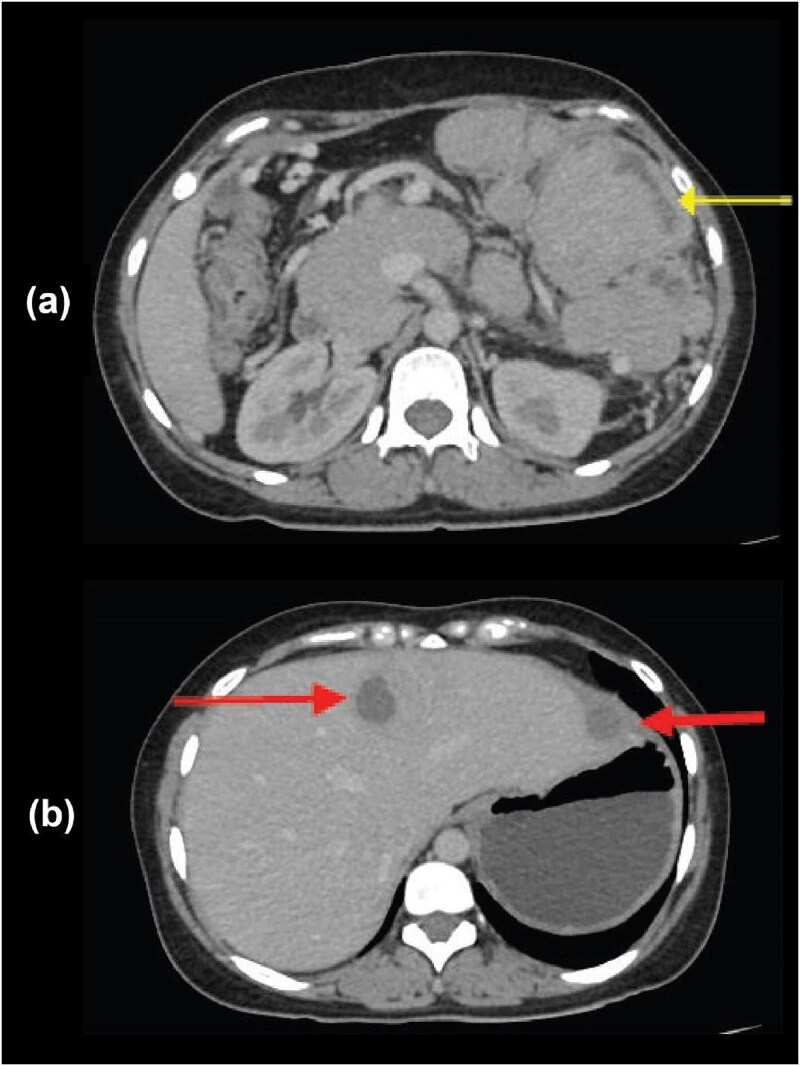
Follow-up CECT of the abdomen in the portal venous phase. (a) Axial image demonstrating multiple large, heterogeneously enhancing omental deposits with areas of central necrosis (arrow). (b) Axial image revealing multiple hypodense hepatic metastases (arrows).

Six months later, she re-presented with recurrent abdominal pain and distension. Repeat CECT demonstrated multiple heterogeneously enhancing omental and mesenteric lesions with necrosis, and metastatic deposits in liver segments III, IVA, and VII (Fig. 4). Ultrasound-guided biopsy of a hepatic lesion revealed pleomorphic small round cells in sheets (Fig. 3b), with diffuse membranous CD99 and nuclear NKX2.2 positivity (Fig. 3c–d), Ki-67 index ~25%, and negativity for CD45, synaptophysin, β-catenin, and desmin. Cytogenetic analysis confirmed the t(11;22)(q24;q12) translocation.

The final diagnosis was metastatic extraosseous ES primary to the pancreas. Given advanced disease in an adult patient, anthracycline-based chemotherapy (doxorubicin 60 mg/m^2^ and dacarbazine 750 mg/m^2^ every 3 weeks) was initiated, yielding disease stability for 7 months. Subsequent ascites and performance status deterioration prompted treatment cessation; the patient died 8 months after chemotherapy commencement.

## Discussion

Primary pancreatic ES is exceedingly rare, with systematic reviews through 2025 documenting only 51–89 confirmed cases [4, 5]. It predominantly affects children, adolescents, and young adults (median age ~23 years; range 2–78 years), with slight male predominance, though well-reported in young females [1]. Typical presentations include abdominal pain (63%–74%), weight loss, and palpable mass, as observed here. Jaundice is common with head involvement (~57% of cases), whereas body/tail tumors often attain large size before symptomatic detection [6, 7].

Radiologically, these tumors manifest as large (>10 cm), heterogeneous solid-cystic masses with necrosis, hemorrhage, and variable enhancement—features mimicking solid pseudopapillary neoplasm, pancreatoblastoma, or neuroendocrine tumors [10]. This overlap frequently precipitates misdiagnosis, as occurred initially in our patient.

Histologically, the small round blue cell morphology with PAS-positive glycogen and occasional rosettes is suggestive but nonspecific [11]. Immunohistochemistry is pivotal: diffuse membranous CD99 (>95% sensitivity) and nuclear NKX2.2 (high specificity) are characteristic, with negativity for neuroendocrine markers aiding differentiation [12]. Definitive diagnosis requires molecular confirmation of EWSR1 rearrangement (most commonly EWSR1-FLI1 via t(11;22)) [13].

In this case, an incomplete initial panel (WT1, cyclin D1, CD10 positivity) erroneously suggested endometrial stromal sarcoma, illustrating the hazards of limited testing. Delayed diagnosis upon metastatic recurrence emphasizes the imperative for early inclusion of CD99, NKX2.2, and molecular studies in young patients with atypical pancreatic masses.

Standard therapy for localized ES presumes micrometastatic disease and thus involves neoadjuvant multi-agent chemotherapy (vincristine-doxorubicin-cyclophosphamide alternating with ifosfamide-etoposide), followed by local control via resection aiming for R0 margins and adjuvant chemotherapy [4]. In this case, upfront surgery achieved microscopic complete resection, but misdiagnosis and loss to follow-up prevented timely administration of Ewing-specific systemic therapy. Metastatic recurrence subsequently required palliative anthracycline-dacarbazine chemotherapy according to adult soft tissue sarcoma protocols [14].

Localized pancreatic ES yields ~70% 5-year survival with multimodal therapy, but metastatic disease confers <20% survival [6]. Adverse factors—large tumor size (>8 cm), delayed treatment, and metastasis [15] —were all present here. This case underscores the profound impact of prompt molecular diagnosis on outcomes in this aggressive yet potentially curable malignancy.

## Data Availability

Data will be made available on reasonable request.
